# Case Report: Primary cutaneous apocrine carcinoma mimicking breast carcinoma - a rare diagnostic challenge

**DOI:** 10.3389/fonc.2025.1664122

**Published:** 2026-02-02

**Authors:** Masih Kameh Khosh, Tobias Hirsch, Hans-Joachim Schulze, Cornelius Kuhnen, Sascha Wellenbrock

**Affiliations:** 1 Department of Plastic and Reconstructive Surgery, Muenster University Hospital, Muenster, Germany; 2 Department of Plastic, Reconstructive and Aesthetic Surgery, Hand Surgery, Hornheide Specialist Clinic, Muenster, Germany; 3 Department of Dermatology, Hornheide Specialist Clinic, Muenster, Germany; 4 Department of Pathology, Clemens Hospital Muenster, Muenster, Germany

**Keywords:** apocrine carcinoma, misdiagnosis, multidiscipinary collaboration, breast cancer, rare entities, histopathology

## Abstract

**Background:**

A 60-year-old male patient presented to a senological clinic with a left axillary tumor. The histomorphological characteristics were ambiguous, initially pointing to apocrine carcinoma of mammary origin. This suspicion led to a delay in establishing the correct diagnosis. Only after complete tumor excision and comprehensive work-up in a multidisciplinary dermato-oncological clinic could a definitive diagnosis of primary cutaneous apocrine carcinoma (PCAC) be reached, allowing for appropriate therapy to be commenced.

**Case Presentation:**

The patient had an unremarkable medical history and had presented for excisional biopsy of an axillary nodule. Following the cancer diagnosis, several diagnostic tests and histopathological evaluations were initially performed in a senological setting to assess the likelihood of (metastatic) mammary carcinoma. Workup included breast ultrasonography, CT and MRI imaging and bone scintigraphy. A previously unrecognized subcutaneous tumor mass was identified in the left axilla, located deep to the excised nodule. No further lesions, either mammary or otherwise, were detected. The patient was referred to a dermato-oncological clinic, and the subcutaneous mass, containing metastatic lymph nodes, was completely excised with clear margins. Following extensive histopathological analysis, imaging and deliberation in the interdisciplinary tumor board, mammary carcinoma and metastatic disease were ruled out and the diagnosis of primary cutaneous apocrine carcinoma was reached. Adjuvant locoregional radiotherapy could subsequently proceed.

**Conclusion:**

This case underscores the importance of an interdisciplinary approach in diagnosing axillary neoplasms and illustrates the valuable role of a high-volume, multidisciplinary skin cancer center in optimizing patient management. Although patients may initially present to a senological clinic, maintaining a broad differential diagnosis is crucial, especially when treating tumors with overlapping or atypical features. This prevents misdiagnoses, ensuring timely and effective care, thereby improving patient outcomes.

## 1 Introduction

Primary cutaneous apocrine carcinoma (PCAC) is a rare malignant tumor originating from adnexal structures, such as sweat glands (1, 2). It is an invasive, locally destructive neoplasm spreading from the reticular dermis to the subcutaneous tissue. Previous reports indicate that it is primarily a malignancy of adults, with no association found with race or sex (3). Although they can develop on any area of the skin, they are more commonly found in regions with a high concentration of apocrine glands, especially the axilla (1). These entities can represent a diagnostical challenge due to clinical variability and lack of consensus on defined histopathological criteria. Moreover, they may display morphological mimicry to other types of cancer with apocrine features, such as breast adenocarcinoma (4). This case study describes an initial misdiagnosis of PCAC as breast cancer, highlights a potentially unique pathoetiological origin, and outlines the subsequent steps leading to the correct diagnosis and appropriate treatment. This report is prepared in accordance with the CARE guidelines.

## 2 Case description

A 60-year-old Caucasian male presented for excisional biopsy of a painless, solitary axillary nodule with slow progressive growth over several months. He had undergone excision of a histologically confirmed syringoma in the same area years earlier, and the current lesion was initially presumed to be a benign recurrence. The patient had an otherwise unremarkable medical and family history.

Histology revealed fully matured squamous epithelium superficially, with deeper layers showing an infiltrative neoplasm composed of epithelial nests exhibiting cribriform to adenoid architecture and, in deeper regions, areas of solid growth. There was focal infiltration into subcutaneous adipose tissue and perineural sheath involvement. The epithelial clusters were lined by cuboidal cells with a high nuclear-cytoplasmic ratio, prominent nuclear membranes, small nucleoli, and no evidence of keratinization or definitive follicular differentiation. Immunohistochemistry demonstrated nuclear androgen receptor expression (90%) and cytoplasmic positivity for CK7, BerEp4 and EMA. CD117 was negative, as were p40, estrogen receptor (ER) and progesterone receptor (PR). HER2/neu showed a score of 2 +. The lesion was identified as a moderately differentiated apocrine carcinoma, with tumor cells extending to the resection margins.

Based on its immunohistochemical profile, the lesion was initially interpreted as triple-negative breast cancer, potentially arising from accessory mammary tissue due to its localization or representing metastasis. The patient was thus referred to a breast clinic for further evaluation.

## 3 Timeline of clinical events

## 4 Diagnostic assessment and therapy

**Figure 1:**
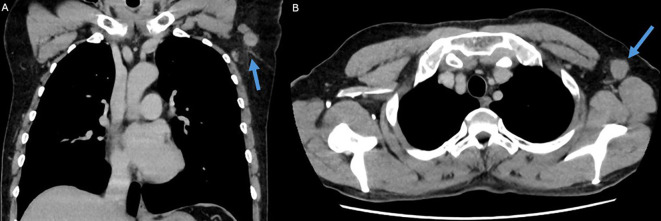
CT imaging demonstrating a tumor with adjacent nodal involvement in the left axilla, located deep to the previously excised skin nodule. **(a)**. Coronal view **(b)**. Axial view.

Comprehensive imaging in a senological setting, including breast ultrasound and CT imaging of the trunk and abdomen, uncovered a previously unrecognized subcutaneous tumor mass in the left axilla, located deep to the site of excision (**Figure 1**). A primary lesion within the breast was not detected. A broad range of imaging studies was therefore conducted to rule out other potential internal primary adenoid tumors, with MRI, gastroscopy, colonoscopy and bone scintigraphy showing no evidence thereof.

Given the clinical and histological ambiguity, which resulted in a delay of approximately 8 weeks, the patient was referred to a dermato-oncological clinic for further evaluation and surgical treatment. There, the subcutaneous axillary mass was excised and further histopathological examination was performed.

**Figure 2:**
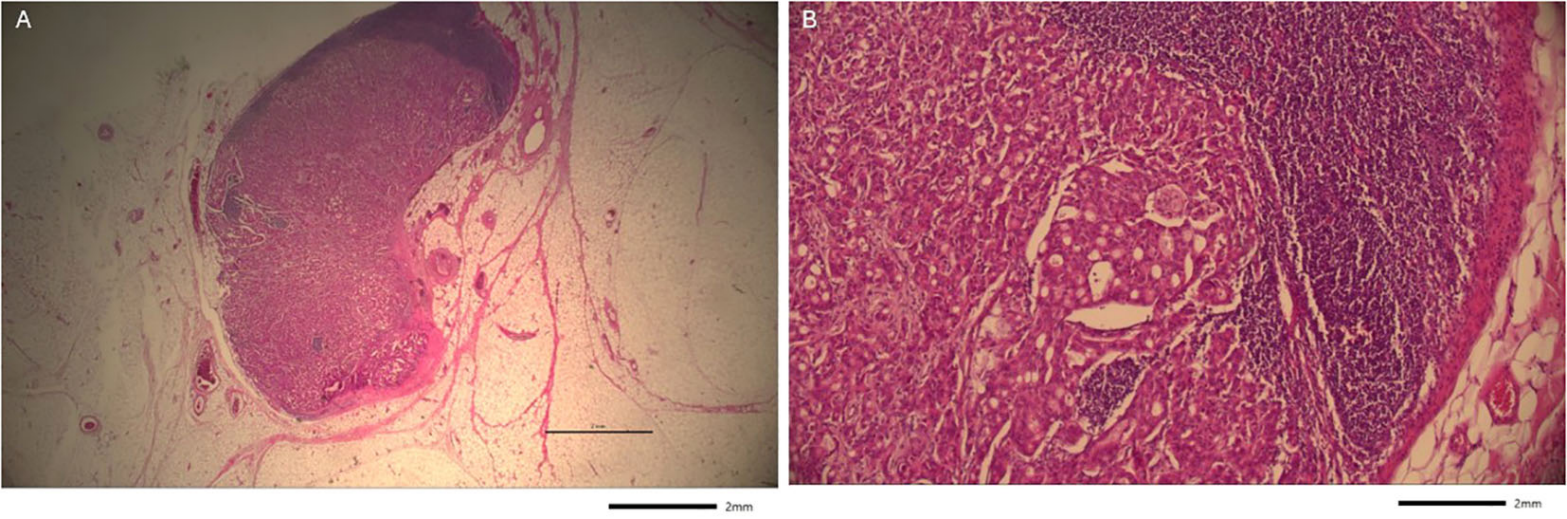
Lymph node metastasis (Hematoxylin and eosin staining). **(a)** Overview of apocrine tumor cells form pale eosinophilic cell clusters, demarcated from the remaining basophilic lymph node parenchyma (1.25x). **(b)** Greater magnification of the same lymph node metastasis (10x).

**Figure 3:**
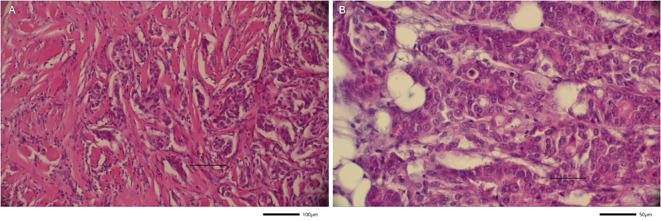
Tumor microscopy (Hematoxylin and eosin staining). **(a)** Trabecular tumor cell clusters in nested patterns with infiltration of adipose subcutaneous tissue (20x). **(b)** Tumor cell complexes and sclerotic tumor matrix in central tumor areas (40x).

The resected neoplastic conglomerate measured 6.6 x 5.9 x 3.5 cm and contained two metastatic lymph nodes (**Figures 2**, **3**). Histological examination confirmed negative tumor margins with a minimum clearance of 1.1 cm in all directions. As the prior immunohistochemical HER2 score of 2+ was deemed equivocal, additional analysis using chromogenic *in situ* hybridization (CISH) was performed to assess HER2 gene amplification, which was negative. Based on histopathological findings, including asymmetric overall architecture, progressively smaller and less dilated ductal structures, and intratumoral perineural invasion, a diagnosis more consistent with a primary tumor than with a cutaneous metastasis of an adenoid carcinoma was supported. A PET-CT scan was additionally conducted, which conclusively ruled out any internal primary disease. The slow progression over several weeks, along with imaging findings, further argued against (metastatic) mammary carcinoma.

After discussion in the tumor board, breast cancer was ruled out, and a diagnosis of primary cutaneous apocrine carcinoma (pT3N1M0, G2) was reached. Although histological margins were clear and no residual disease was detected clinically or radiologically, adjuvant locoregional radiotherapy and close surveillance were initiated following interdisciplinary discussion to manage the risk of regional recurrence. **Table 1** summarizes the timeline of clinical events.

**Table 1:** Clinical timeline.

Date	Clinical event
Mar 2021	Initial excision of left axillary nodule; diagnosed as syringoma.
Jan 2024	Patient noticed a new nodule in the left axilla.
Jun 2024	Nodule excised; referred to senology clinic due to suspected breast cancer.
Jul 2024	Extensive imaging and histopathological testing in senology clinic.
Jul 2024	Referral to dermato-oncological clinic for further work-up.
Aug 2024	Complete excision of subcutaneous left axillary tumor mass.
Sep 2024	Diagnosis determined in tumor board; referred for adjuvant radiotherapy.
Oct 2024	Locoregional adjuvant radiotherapy initiated

The patient was in good health and showed no clinical or radiological evidence of recurrence one year after excision of the axillary node.

## 5 Discussion

The marked histological similarity between PCAC and breast adenocarcinoma often precludes definitive distinction based on microscopic features alone (2, 5). Although CK7 positivity is suggestive of cutaneous apocrine carcinoma, it lacks specificity, as CK7 is widely expressed in various epithelial malignancies, including those of lung and ovary (6). Therefore, CK7 should be interpreted only in combination with additional markers and within the clinical context to avoid misclassification. The absence of estrogen, progesterone and HER2 receptor expression may also argue against a diagnosis of breast cancer, though it does not rule out triple-negative breast carcinoma (2).

Nonspecific markers may be expressed across a wide range of carcinomas and other neoplasms, limiting their value for definitive classification and contributing to diagnostic uncertainty, particularly in poorly differentiated or undifferentiated tumors, where classic morphologic features are absent or equivocal (7, 8). In the absence of pathognomonic immunostains, broad immunohistochemical panels are often required, which can deplete limited tissue samples and still fail to yield a conclusive diagnosis in some cases (9). In addition, technical variables, interpretive subjectivity, and aberrant antigen expression further increase the risk of diagnostic pitfalls and misclassification (10).

A prior benign skin lesion may offer an additional diagnostic clue, as malignant transformation has been reported (11, 12). In the present case, the occurrence of PCAC in the same region as a previously excised syringoma raises the question of whether the carcinoma may have arisen from the earlier lesion. However, considering the distinct histogenetic origins of eccrine and apocrine glands, the development of apocrine carcinoma from a benign eccrine tumor is unlikely and may suggest that mixed apocrine differentiation was unrecognized at that time.

Furthermore, distinguishing PCAC from breast cancer - whether occult metastasis or arising from accessory mammary tissue - requires both clinical evaluation and comprehensive imaging. Accessory breast tissue can be found along the embryonic milk line, which extends bilaterally from the axilla to the groin. Cancers arising in these areas may therefore be misattributed to breast origin based on location alone, which is particularly relevant considering the axilla is the most frequent site of primary apocrine apocrine carcinoma (1). Primary internal adenocarcinomas that have metastasized to the skin typically indicate advanced disease and poor prognosis, whereas PCAC usually follows a more indolent course. Prognosis is chiefly influenced by the presence of lymph node metastases and the extent of cellular anaplasia, with the absence of nodal involvement conferring a significant survival benefit (1).

The rarity of the disease has prevented the establishment of standardized treatment protocols. Wide local excision is generally considered the treatment of choice. Although regional lymph node involvement is frequently observed, there is no consensus on the role of (radical) lymphadenectomy in reducing the risk of disease recurrence (1, 3, 13). Adjuvant radiotherapy may offer benefit in moderate to poorly differentiated lesions with more advanced locoregional involvement (1, 12, 14). Data regarding systemic therapy in disseminated disease is limited, with some studies demonstrating potential benefit from chemotherapy or endocrine treatments, including anti-androgen therapy (15–18).

A lack of distinctive clinical features, slow progression, and histological ambiguity may result in a substantial delay before appropriate therapy is initiated (3). This case underscores the importance of maintaining a broad differential diagnosis when evaluating tumors with non-specific or overlapping clinico-pathological features. Clinicians, particularly senologists, should consider the possibility of PCAC when assessing a suspected cutaneous manifestation of triple-negative breast carcinoma. Recognizing this distinction is crucial to avoid misdiagnosis, which could lead to unnecessary surgery or inappropriate systemic therapy. Accurate identification ensures timely, appropriate treatment and ultimately improves patient outcomes.

## 6 Patient perspective

### 6.1 Translated from German

“I went to see my doctor because I had noticed a small, slowly growing lump in my left armpit. A few years earlier, the doctor had removed something similar from nearly the same spot, which had turned out to be harmless. So, at first, we both assumed it was just another benign recurrence.

However, after some time, my doctor said that it might be cancer. Naturally, I was deeply unsettled, but I tried not to worry too much while we waited for further tests. Following these, they told me it could be metastatic breast cancer, which completely devastated me. After a while, they suggested it might instead be a form of skin cancer instead and referred me to your clinic for further treatment.

This whole experience felt like an emotional rollercoaster. The conflicting diagnoses caused me a lot of stress and many sleepless nights. I have a physically active job and exercise regularly, so I always felt strong and energetic. But as I started hearing these different possible diseases, I became both physically and emotionally exhausted. When they finally told me that no internal tumors had been found, I began to feel some relief. Still, the changing opinions about my tumor were incredibly draining, and it was this uncertainty that weighed on me the most. I was grateful that all the doctors, including the chief medical director, were so engaged and took such a personal interest in my case.

I feel good now, but I’ve lost a certain carefree attitude towards my health. I have always lived a healthy lifestyle — I eat well, don’t drink or smoke, and don’t take any medications. I never imagined something like this could happen to me, but that sense of security is gone. There is always a small thought in the back of my mind that it could come back one day. Nevertheless, I try not to dwell on it and instead focus on enjoying each day to the fullest.”

## Data Availability

The original contributions presented in the study are included in the article/supplementary material. Further inquiries can be directed to the corresponding author.
